# A new strain of *Volutella citrinella* with nematode predation and nematicidal activity, isolated from the cysts of potato cyst nematodes in China

**DOI:** 10.1186/s12866-021-02385-x

**Published:** 2021-11-22

**Authors:** Xinyue Zhang, Hui Zhang, Zhaochun Jiang, Qing Bai, Shishi Wu, Yong Wang, Cheng Li, Xiangyu Zeng, Xiuhai Gan, Xin Xie, Zhong Li, Zaifu Yang

**Affiliations:** 1 Department of Plant Pathology, College of Agriculture, Guizhou University, Guiyang, Guizhou China; 2 Guizhou Station of Plant Protection and Quarantine, Guiyang, Guizhou China; 3 Center for Research and Development of Fine Chemicals, Guizhou University, Guiyang, Guizhou China

**Keywords:** Biocontrol, Nematophagous fungi, Plant parasitic nematode, *Volutella citrinella*

## Abstract

**Background:**

Plant parasitic nematodes (PPNs) are responsible for causing many plant diseases and are extremely difficult to control at present. Currently, due to the negative effects of chemical agents on the environment and human health, the development of new biological pesticides has become an important part of plant nematode control. Nematophagous fungi refers to a class of fungi that kill plant nematodes. Notably, a large number of nematophagous fungi resources remain to be studied. The objective of our study was to use in vitro screening to identify nematophagous fungi and select strains that were highly active against nematodes, providing a primary research for the development and utilization of new nematophagous fungi.

**Results:**

A new nematophagous fungal strain (GUCC2219) was isolated from cysts of possibly *Globodera* spp. and *Heterodera* spp., identified as *Volutella citrinella*. The hyphae of *V. citrinella* produced ring structures of variable size and exhibited predatory and nematicidal activity. The hyphal predation rates (in vitro) against three species of nematodes, *Aphelenchoides besseyi*, *Bursaphelenchus xylophilus*, and *Ditylenchus destructor*, averaged 59.45, 33.35, and 50.95%, respectively, while the fermentation broth produced by the fungus exhibited mortality rates of 100, 100, and 55.63%, respectively, after 72 h.

**Conclusion:**

*V. citrinella* is a new strain with nematophagous properties, which are a novel discovery. At the same time, this is the first report of nematicidal and nematode predation activity in the genus *Volutella*.

**Supplementary Information:**

The online version contains supplementary material available at 10.1186/s12866-021-02385-x.

## Background

Plant parasitic nematodes (PPNs) are major pathogens of many crops and comprise more than 4100 species globally [1]. Nematodes feed on a wide range of important agricultural crops, including wheat, rice, soybeans, potatoes, tomatoes and sugar beets [2]. A variety of aboveground symptoms become evident after nematodes have infected the root system of a plant, such as leaf discoloration, leaf spot, wilting, stunted growth, and increased susceptibility to other pathogens [2–4]. Plant diseases caused by nematodes have gradually become a main limiting factor for crop yield and quality and have been the cause of significant economic losses ($80 billion/year) globally [5–7].

Species of plant nematodes that impact agriculture include cyst nematodes (*Globodera* and *Heterodera* spp.), root knot nematodes (*Meloidogyne* spp.), foliar nematodes (*Aphelenchoides* spp.), pine wilt nematode (*Bursaphelenchus xylophilus*), *Ditylenchus destructor*, *Anguina tritici* [8–10]. Among them, *A. besseyi*, *B. xylophilus* and *D. destructor* are especially important in China. *Aphelenchoides besseyi* mainly damages rice, whose leaves exhibit white tips after becoming infected and then become necrotic. The flagged leaves are curled and deformed, inflorescences are reduced in size and the heads do not develop mature grains, resulting in a decrease in yield [11]. Yield can be reduced in severe cases by as much as 60% [12]. In fact, *A. besseyi* has been listed as one of the ten most harmful plant nematodes due to its potential impact on yield [13]. *Bursaphelenchus xylophilus* is responsible for causing many tree diseases globally [14] and is classified as a quarantine pathogen by most countries in the world [15]. It is also listed among the ten most harmful plant nematodes [13]. It is mainly spread by the beetle *Monochamus alternatus* feeding on healthy pine trees [16]. When the pine wood nematode enters the stem of pine trees, it migrates through resin canals feeding on parenchyma cells, ultimately killing the host tree [17]. It is responsible for causing hundreds of millions of dollars in economic losses globally, including in Japan, the United States, and Canada [18, 19]. *Ditylenchus destructor* is also one of the most destructive plant pathogenic nematodes [20]. It can parasitize more than 120 host plants in China and is one of the main pathogens of potatoes and sweet potatoes [21], causing a 20 to 50% yield reduction and even 100% crop loss in endemic areas [22]. In summary, the economic losses caused by the three plant nematodes distributed to China and even the whole world should not be underestimated. At the same time, they damage plant leaves; the inside of plants and plant tubers respectively. So, they can be used as representative PPNs that damage different parts of plants. Therefore, the effective control methods for these three plant nematodes may also be applicable to other plant nematodes.

Due to the negative effects of chemical agents on the environment and human health, certain chemical agents have been recently restricted or banned [23], and thus, the development of new biological pesticides has become an important part of plant nematode control [24]. Nematophagous fungi refers to a class of fungi that have the ability to parasitize, capture, colonize, and produce toxins that kill plant nematodes [25]. To date, more than 700 species of nematophagous fungi have been reported around the world [26], which were distributed multiple genera (Additional file 1: Schedule 1). Although there are so many types, there are few commercial products that have been successfully developed into biocontrol preparations, and the number of biocontrol preparations used in production is not many [27]. In addition, a large number of nematophagous fungi remain to be discover in nature [28, 29]. Therefore, the excavation of new nematophagous fungi is of great significance to natural resources and the development of biological pesticides.

Approximately 380 species of nematode-trapping fungi have been reported from different regions of the world [25]. However, there is no report about the fungi of the genus *Volutella* as nematode-trapping fungi. *Volutella* is a widespread genus of the Nectriaceae family and approximately 120 described species of *Volutella* have been identified from various parts of the world [30]. The genus *Volutella* is poorly researched despite the common occurrence and broad distribution of these species [31]. According to reports, *V. citrinella* (*Stilbella aciculosa* [32]) oxidizes Mn (II) to Mn oxides by producing extracellular superoxide during cell differentiation and this microbial extracellular superoxide production may play a central role in the cycling and bioavailability of metals (e.g., Hg, Fe, Mn) and carbon in natural systems [33]. In addition, Osono and Takeda [34] reported that *V. ciliata* plays an important role in plant litter decomposition in forest ecosystems through soil nutrient recycling and the accumulation of organic matter in soil. However, other species of the genus can cause a variety of plant diseases, such as *Volutella pachysandricola* causing Volutella blight (sometimes called leaf blight and stem canker) on Japanese pachysandra (*Pachysandra terminalis*) [35]; *V. buxi* causing Boxwood Volutella stem blight or canker on boxwood (*Buxus* spp.) [36]. There were beneficial microorganisms and plant pathogens in the fungus of the genus *Volutella*, which are worthy of further study.

In the present research, colonizing microorganisms were isolated from cysts of possibly *Globodera* spp. and *Heterodera* spp. of potatoes growing in Weining County, Guizhou Province, China. A total of 147 isolates were obtained, mainly fungi. Their nematicidal properties were evaluated by in vitro screening. One isolate (GUCC2219) was found to exhibit high hyphal predation rates in vitro against *Aphelenchoides besseyi*, *Bursaphelenchus xylophilus*, and *Ditylenchus destructor,* with predation rates averaging 59.45, 33.35, and 50.95%, respectively, while its fermentation broth exhibited mortality rates of 100, 100, and 55.63%, respectively, after 72 h. The GUCC2219 fungal isolate was identified as *Volutella citrinella* based on morphological observations and phylogenetic analysis of the genetic sequence of the internal transcribed spacer (ITS) and large subunit (LSU) regions of DNA. This is the first report of nematicidal and nematode predation activity in the genus *Volutella.*

## Results

### Fungi identification (GUCC2219)

**Fig. 1:**
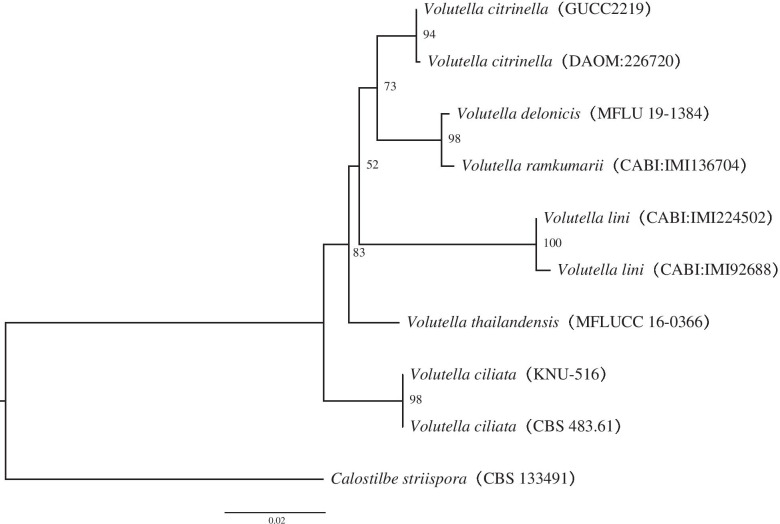
Phylogenetic tree generated from RAxML analysis based on combined LSU and ITS sequence data of *Volutella. citrinella* (GUCC2219). *Calostilbe striispora* (CBS 133491) was designated as the outlier taxon. The scale bar indicates 0.02 nucleotide changes per site

The sequences of the polymerase chain reaction (PCR) products obtained from strain GUCC2219 were uploaded to GenBank and subjected to Basic Local Alignment Search tool (BLAST) analysis. The highest similarity was obtained to accession numbers MZ148447 (ITS) and MZ148449 (LSU). In the phylogenetic tree (Fig. 1), GUCC2219 clustered with the type culture of *Volutella citrinella* (DAOM: 226720) and exhibited high sequence similarity, 99% (530/531) and 100% (877/877) to its ITS and LSU gene regions, respectively.

**Fig. 2:**
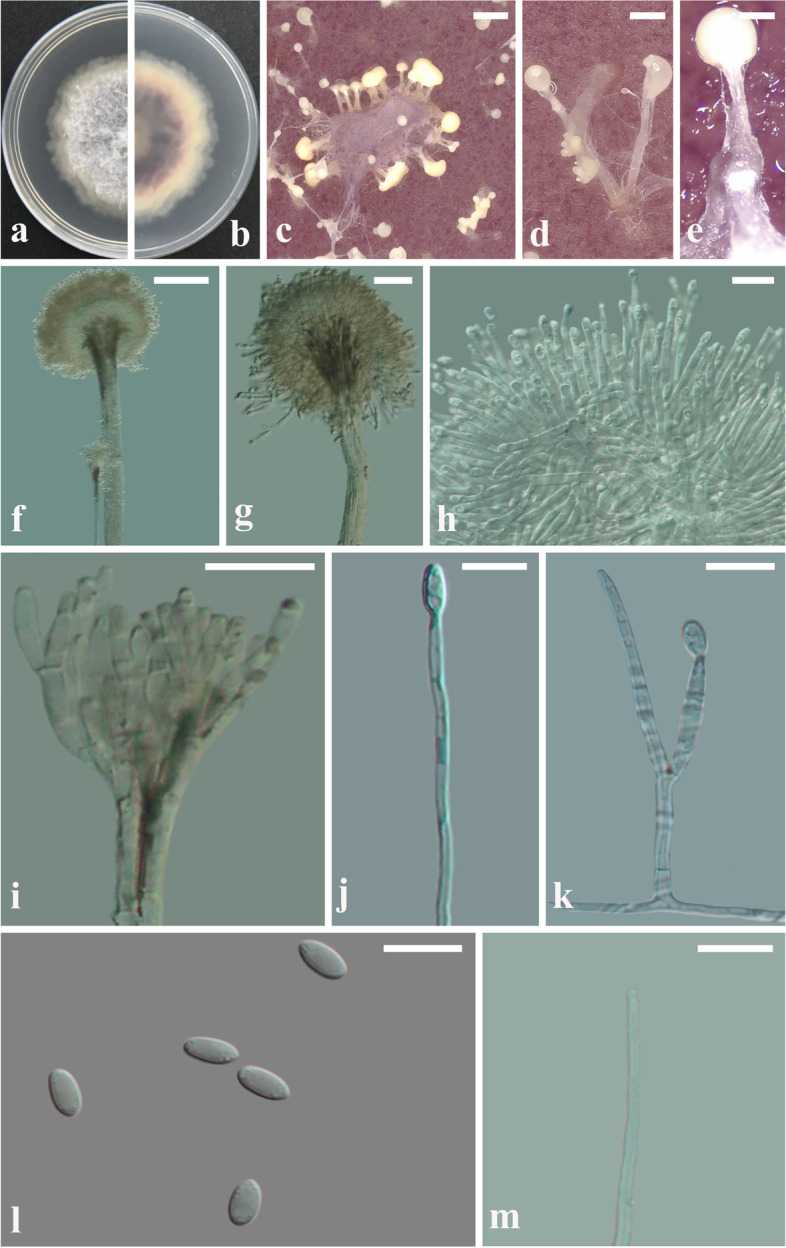
Colony and microscopic features of *Volutella. citrinella* (GUCC2219). **a**, **b**. Top and bottom images of GUCC2219 cultured on PDA for 15 d. **c**, **d**, **e**. Purple pigment and yellowish slime of the synnemata of colonies growing on CMA. f-k. Conidiogenous cells. l. Conidia. m. Seta-like marginal hypha in culture. Scale bars: c = 1000 μm, d, e = 100 μm, f, g, i, j = 50 μm, h, k, l, m = 10 μm

Morphological observations were made after the GUCC2219 strain was cultured on potato dextrose agar (PDA) and cornmeal agar (CMA) for 14 days at 25 °C. Observations indicated that colonies were superficial on PDA, reaching 20-30 mm diam. A purple-red pigment was produced on the underside of the colony (Fig. 2a, b). More purple-red pigment was evident when GUCC2219 was cultured on CMA than when it was cultured on PDA. Conidial masses that formed on the surface were yellow-white. Synnemata were lightly colored, 350-1300 μm tall, and 26-40 μm wide (Fig. 2c, d, e). Conidiogenous cells and phialides were 12-20 (length) × 2-3 μm (width) (Fig. 2f-k). Conidia were ellipsoidal to oblong-ellipsoidal and 4.5-7 (length) × 2-3.5 (width) μm in size (Fig. 2l). Marginal hyphae were verrucose near the capitulum (Fig. 2m). No aerial mycelia or chlamydospores were observed on PDA or CMA. These morphological characteristics were somewhat different from those described for *V. citrinella* by Rezakhani et al. [37]., who described the size of synnemata as 300-1450 μm tall and 25-50 μm wide and the size of the conidiogenous cells and conidia as 13-25 × 1.5-2 μm and 3-5 × 1-2 μm, respectively. Despite these differences, however, we felt that the combined morphological observations and phylogenetic analysis confirmed isolate GUCC2219 to be a strain of *V. citrinella*.

### In vitro predatory activity of *V. citrinella* (GUCC2219) against nematodes

**Fig. 3:**
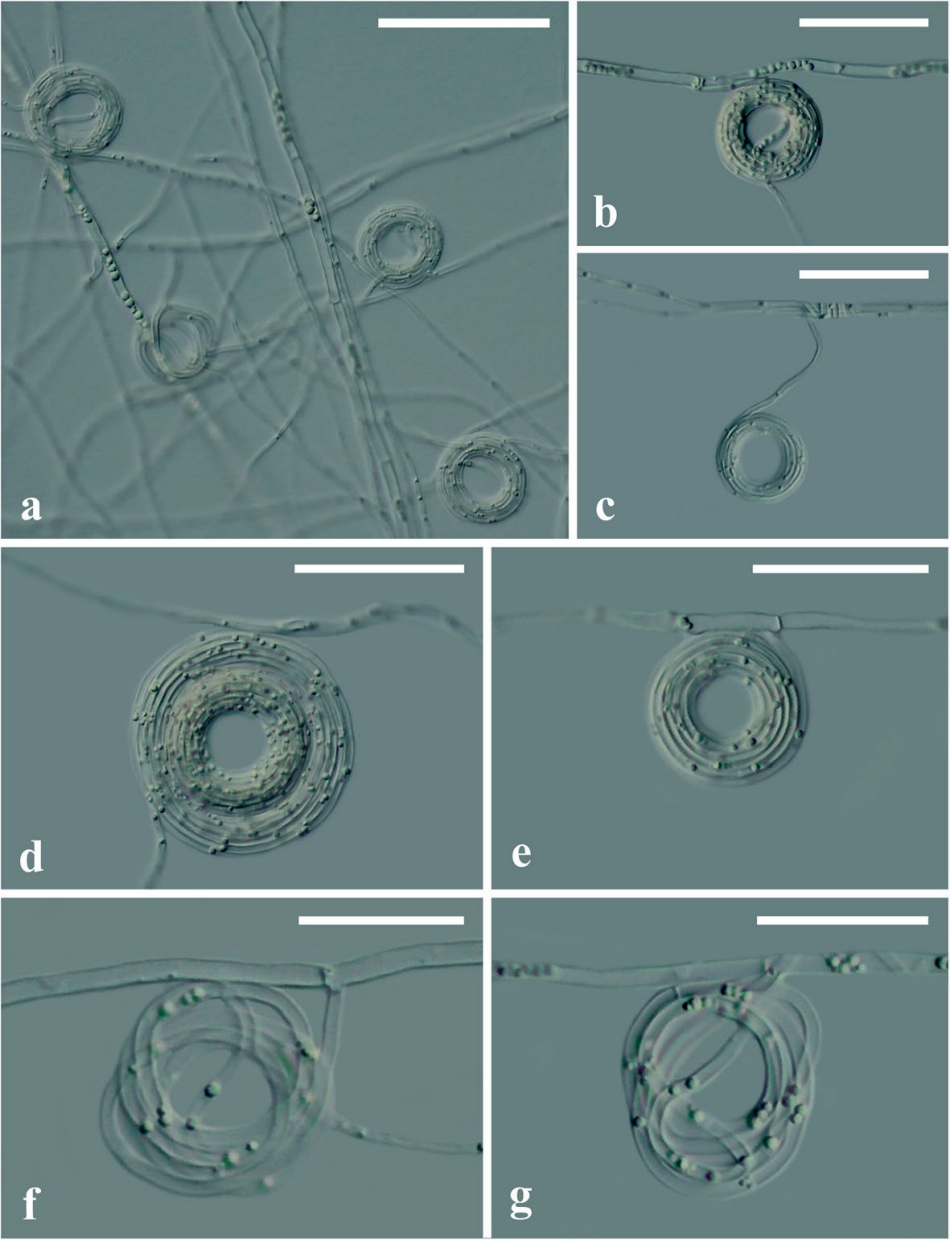
**a**. Ring structures of *Volutella. citrinella* (GUCC2219) cultured on water agar (WA). **b**, **c**. Small, regular-shaped ring structures. **d**, **e**. Large regular-shaped ring structures. **f**, **g**. Irregular-shaped ring structures. Scale bars = 50 μm

Microscopic examination of *V. citrinella* (GUCC2219) at 1% water agar (WA) revealed that it produced a large number of hyphal rings through the twining of mycelia (Fig. 3a). There were three types: small, regular-shaped (Fig. 3b, c), large regular-shaped rings (Fig. 3d, e), and irregularly shaped rings of various sizes (Fig. 3f, g). Measurements of the rings indicated that the average diameters of the outer and inner rings were 26.40 μm and 12.24 μm, respectively. The average thickness of mycelial twining was 7.75 μm. Sizes were based on the measurement of 100 randomly sampled rings (Table 1).

**Table 1:** Diameter (μm) of inner and outer rings and thickness of individual hyphae making up the ring structures produced by *Volutella citrinella* (GUCC2219)

Ring size	Mean (μm)	Range (μm) (***n*** = 100)
Diameter of outer ring	26.40 ± 6.69	15.69-61.89
Diameter of inner ring	12.24 ± 3.03	6.61-33.20
Thickness of mycelium winding	7.75 ± 2.57	4.27-22.11

^a^Data represent the mean ± SE (*n* = 4)

**Fig. 4:**
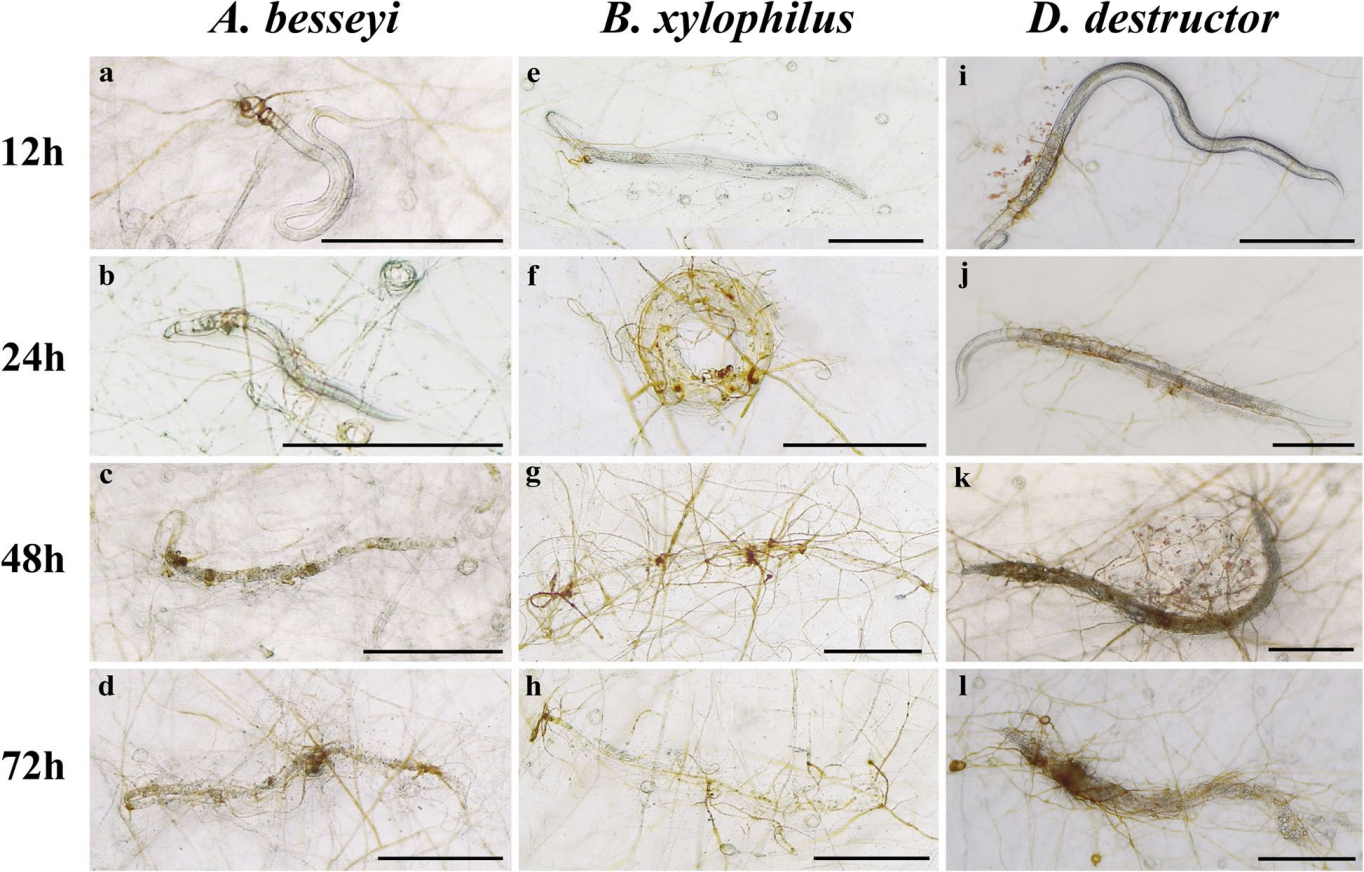
Predatory activity of *Volutella. citrinella* (GUCC2219) against three plant pathogenic nematodes on water agar (WA). **a**-**d**. Predatory activity against *A. besseyi* at 12, 24, 48, and 72 **h**; **e**-**h**. Predatory activity against *B. xylophilus* at 12, 24, 48, and 72 h; **i**-**l**. Predatory activity against *D. destructor* at 12, 24, 48 and 72 h). Scale bars = 200 μm. Data represent the mean ± SE (*n* = 4)

The quantity of the rings did not increase after the fungal cultures were inoculated with nematodes. Nematodes moved freely on WA plates of GUCC2219 cultures during the first 12 h of coincubation, and little change occurred in the morphology of the rings. Between 12 and 24 h, however, the movement of the nematodes began to become limited, and the rings turned a golden color when a nematode became entrapped. Nematodes became entangled and completely entrapped (could not move) due to their increased entanglement with hyphae after 24-48 h of coincubation. Two days later, the hyphae had started to attach to the surface of entrapped nematodes and completely engulf them. After 72 h of coincubation, the number of fungal mycelia composing the rings increased, the body wall of the entrapped nematodes dissolved, the internal contents of the nematodes consumed, and only traces of the entrapped nematodes remained (Fig. 4a-l).

Under in vitro conditions, the GUCC2219 strain of *V. citrinella* was cultured on 1% WA and separately coincubated with three different species of nematodes. The number of total nematodes entrapped in five independent microscopic fields of view was recorded over 72 h. The results indicated that *V. citrinella* (GUCC2219) exhibited significant predatory activity against the three species of nematodes at different points in time. In general, the percentage of trapped nematodes increased with the time of coincubation for all three species of nematodes (Table 2). The predation activity of *V. citrinella* (GUCC2219) was greatest against *A. besseyi,* with an average percentage of 59.45% at 72 h, followed by *D. destructor* and *B. xylophilus*, with averages of 50.95 and 33.35%, respectively (*P* < 0.05).

**Table 2:** The predation rate of *Volutella citrinella* (GUCC2219) against three species of plant pathogenic nematodes on water agar

Nematodes	Treatment	Incubation Time (h)			
		12	24	48	72
*A. besseyi*	Control	3.50 ± 0.50 b	5.50 ± 0.96 b	9.50 ± 1.26 b	10.67 ± 0.67 b
	GUCC2219	13.88 ± 0.87 a	22.37 ± 2.05 a	37.67 ± 3.17 a	59.45 ± 3.76 a
*B. xylophilus*	Control	6.50 ± 0.96 b	7.50 ± 0.50 b	9.50 ± 1.50 b	11.00 ± 1.73 b
	GUCC2219	19.66 ± 2.40 a	27.04 ± 2.19 a	32.40 ± 2.11 a	33.35 ± 0.96 a
*D. destructor*	Control	2.00 ± 1.15 b	3.00 ± 1.00 b	5.00 ± 1.91 b	8.00 ± 1.63 b
	GUCC2219	8.64 ± 1.13 a	17.65 ± 2.34 a	30.98 ± 0.98 a	50.95 ± 2.21 a

*Note: Data represent the mean ± SE (*n* = 4). Different lowercase letters indicate a significant difference (*P* < 0.05) between the control and GUCC2219 strains within a species and within a given time point

### Nematicidal activity of the *V. citrinella* (GUCC2219) fungal fermentation filtrate

**Fig. 5:**
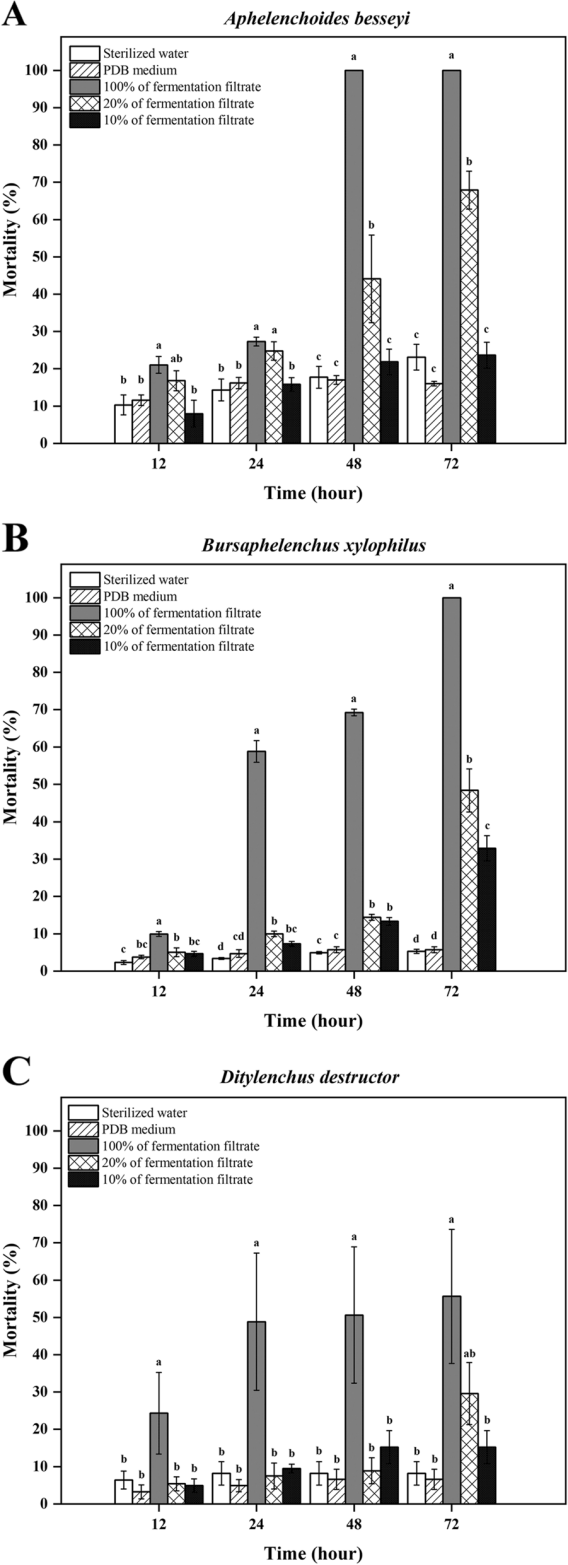
In vitro nematicidal activity of the fermentation filtrate of *Volutella. citrinella* (GUCC2219) against three species of plant pathogenic nematodes (A. *A. besseyi*; B. *B. xylophilus*; C. *D. destructor*). *Note: Different letters indicate a significant difference between the different treatments within a given time point. Lowercase letters indicate significantly different means at *P* < 0.05. Data represent the mean ± SE (*n* = 4)

The nematicidal activity of the *V. citrinella* (GUCC2219) fermentation filtrate was significantly different from that of the potato dextrose broth (PDB) medium alone (Fig. 5), the latter of which exhibited no nematostatic or nematicidal activity as nematode mortality in PDB was statistically similar to that in sterilized water (P < 0.05).

The nematicidal activity of the undiluted fermentation filtrate of *V. citrinella* (GUCC2219) was the highest against *A. besseyi*, producing mortality levels of 21, 27, 100, and 100% at 12, 24, 48, and 72 h, respectively (Fig. 5A). The mortality rate in *Bursaphelenchus xylophilus* also increased rapidly, being 10, 59, 69 and 100% at 12, 24, 48, and 72 h, respectively (Fig. 5B). *Ditylenchus destructor* was the most resistant to the fermentation broth, exhibiting a mortality rate of only 51% at 72 h (Fig. 5C). Collectively, the data indicate that the fermentation broth of *V. citrinella* (GUCC2219) exhibited different levels of nematicidal activity against the three different species of nematodes.

Although the nematicidal activity of the 20% preparation of the fermentation filtrate is significantly lower than that of the 100% fermentation filtrate, it still exhibited the high nematicidal activity against *A. besseyi* (68%), *B. xylophilus* (48%) and *D. destructor* (30%) at 72 h (P < 0.05). (Fig. 5A-C). A 10% preparation of the fermentation broth also exhibited a significant level of nematicidal activity at 72 h, relative to the controls, against *B. xylophilus* (33%) and *D. destructor* (15%) (P < 0.05) (Fig. 5B, C).

## Discussion

Nematode traps are generally produced by fungi under low-nutrient or nutrient-deficient conditions but can also be induced by other agents, such as abscisic acid, amino acids, bacteria, nematodes, etc. [38]. The trap structures formed by *V. citrinella* (GUCC2219) were mainly produced under low-nutrient WA, which mainly form three ring types: small, regularly shaped; large, regularly shaped and irregularly shaped rings of various sizes. There were some differences from currently reported fungal nematode traps, such as adhesive nets, adhesive knobs, adhesive branches, and constricting rings [39]. Nematodes that are preyed upon by *V. citronella*, are entangled by the hyphae and rings. Of interest is the fact that the rings turn a golden color when the nematodes are caught. This feature has not been reported for other fungi that uses the same method of entrapment. We speculate that a chemical substance that are released when a nematode is trapped, might be responsible for the color change.

Previous studies have reported nematicidal activity of the fermentation filtrate of some fungi, such as *Beauveria bassiana* (Snef2621) against *A. besseyi*, exhibiting a mortality rate of 64.85% at 48 h [40]. In contrast, *V. citrinella* (GUCC2219) fermentation filtrates have higher nematicidal activity, and the mortality rate reached 100% at 48 h. Wu et al. [41] reported *Myrothecium verrucaria,* whose fermentation filtrates exhibited a high mortality rate (96.1%) against *B. xylophilus*. In our research, *V. citrinella* (GUCC2219) fermentation filtrates also had high activity against *B. xylophilus*, with a mortality rate of 100%. The study by Zhang et al. [42] showed that *Bacillus subtilis* 1 × 10^9^ spore/g wettable powder (WP) and *Trichoderma* fertilizer ≥2 × 10^8^ spore/g dustable powder (DP) had nematicidal activity against *D. destructor*, the corrected mortality rate reached 73.45 and 44.76% at 72 h, respectively. The activity of *V. citrinella* (GUCC2219) fermentation filtrates against *D. destructor* (51%) was between those of these two microbial agents. The fermentation filtrate of *V. citrinella* (GUCC2219) had nematicidal activity against all three species of nematodes that were also evaluated, although the level of nematicidal activity differed among the three different species. Collectively, our data provide information on a new fungal strain (*V. citrinella*) with nematicidal activity.

Compared with the previously reported commercial preparations of fungi, such as *Paecilomyces lilacinus* and *Verticillium chlamydosporium* [43], they are mainly used as endoparasitic fungi to prevent and control plant nematodes that damage plant roots, such as root knot nematodes [44], and based on In vitro studies have shown that our fungus has nematicidal activity whether it is the fungus itself or its metabolites. It has high nematicidal activity against the three nematodes that destroy the three parts of the plant. Therefore, our fungus is suitable for developing nematicides from the perspective of insecticidal ways and target nematodes. Further studies need to be conducted to the biological characteristics of the fungi and isolate its nematicidal activity substances, at the same time, determining if this is a common feature of species within the genus *Volutella.*

## Conclusions

In the present study, we report a new nematophagous fungal strain (GUCC2219) that was isolated from cysts formed by potato cyst nematodes. The GUCC2219 strain exhibited the potential to control three species of nematodes in vitro, *A. besseyi*, *B. xylophilus*, and *D. destructor*, and the mycelia also changed from pale white to light yellow after capturing a nematode. The GUCC2219 strain was identified as *V. citrinella* based on morphological observations and a phylogenetic analysis of the DNA sequences of its ITS and LSU regions. Our finding represents a new record in China. The fungus was able to produce hyphae that formed complex ring structures capable of trapping the three different species of nematodes that were evaluated. Our results clearly demonstrated the predatory properties of the hyphal rings formed by *V. citrinella* (GUCC2219), representing a new and novel discovery. *V. citrinella* (GUCC2219) produced a constant number of hyphal ring structures in a low-nutrient environment and the fermentation filtrate against the three species of nematodes in a short time without being concentrated, which presumably contributed to its strong predatory activity and nematicidal properties. Our research has laid the foundation for the application of the nematophagous fungi (GUCC2219). According to the results of biological characteristics and nematicidal ways, the biological pesticides can be further developed. One is the spore suspension, and the other is nematicidal fermentation filtrate. For seed-borne diseases such as *A. besseyi*, the seeds can be soaked before sowing, or the inoculant can be sprayed on the leaves and stems of the rice; *B. xylophilus*, which are parasitic nematodes in pine trees, spores or nematicidal fermentation filtrate can be injected into the plants through a syringe; *D. destructor*, which parasitizes mainly tubers, bulbs and root crops, root irrigation can be carried out to achieve the purpose of preventing nematode diseases. Of course, these are only a bold speculation, and further research is needed to prove the applicability of this strain in the field.

## Methods

### Sample collection and fungal strain isolation

Soil samples were collected from 18 fields of potato plants growing in Weining County, Guizhou, China, that were infected by PCNs. In each field, ten plots of a 5 × 5 m grid were selected surrounding infected potato plants, and in each grid, an approximate volume of 250 ml of soil was collected from rhizosphere zone (0-20-cm depth). The individual samples of each plot were collected and mixed in a bucket to obtain a single composite sample [45]. Each composite sample was thoroughly mixed to obtain a homogenous sample. A subsample of an approximate volume of 500 ml soil was then air-dried at 37 °C for 2 days for PCNs cyst extraction [46–48]. Cysts were extracted from a subsample of 100 g of dried soil using the Baunacke method [49, 50], i.e., dried cysts that floated in water were decanted and collected on a 250 μm sieve.

The selected cysts were surface sterilized in 0.2% H_2_O_2_ for 3 min following three washes with distilled water. The surface-sterilized cysts were individually placed onto 1% WA plates. Plates were incubated at room temperature and monitored regularly. Fungal mycelia growing from the cultured cysts were then recultured several times on new PDA plates. The obtained isolates were initially screened for nematode predation activity. A total of 44 isolates were screened (*A. besseyi*), one isolate exhibited nematode predation activity (Attached Fig. 1), which was conserved in the Culture Collection of the Department of Plant Pathology, Agriculture College, Guizhou University (GUCC) and the strain number was GUCC2219. Therefore, this fungal strain was selected for further study.

### Collection and culturing of nematodes

Samples of *A. besseyi* (The specimens were identified by Zaifu Yang) were collected from rice growing in Dushan County, Guizhou Province, China, and *D. destructor* was provided by the Nematode Laboratory, Fujian Agricultural and Forestry University. The nematodes were propagated on carrot callus [51]. The nematodes were removed from the carrot-callus cultures and sterilized with 0.1% streptomycin sulfate for 10 mins, washed three times with double-distilled water, and then cultured on carrot discs at 25 °C for 30 d.

*Bursaphelenchus xylophilus* was provided by the Center for Research and Development of Fine Chemicals, Guizhou University, Guiyang, China. It was cultured on PDA plates containing *Botrytis cinerea* [52], which was grown on PDA plates at 28 °C for 7 d. Nematodes were placed in the developing colony of *B. cinerea*, and the plates were incubated at 28 °C until the *B. cinerea* colony was completely consumed. The nematodes were separated from *B. cinerea* cultured using the Baermann funnel method [50]. A nematode suspension was made at a concentration of 1000 nematodes/ml for use in the predatory and nematicidal activity assays of the fungal isolates.

### Fermentation filtrate preparation (GUCC2219)

For the preparation of PDB medium, 200 g potatoes were added to 1 L of distilled water and boiled for 30 mins. The mixture was then filtered through double gauze, and 20 g dextrose was added to the filtrate. Subsequently, 100 ml aliquots of the prepared PDB medium were placed into 250 ml conical flasks and sterilized in an autoclave at 121 °C for 30 min. A single pure culture of the GUCC2219 isolate was cut into small pieces approximately 5 mm in diameter using a sterilized cutting blade. Five pieces were added into to 100 ml of sterilized PDB medium, which was then placed on a rotary shaker at 28 °C set at 200 rpm for 7 days. Afterwards. the medium was filtered and stored at 4 °C.

### Morphological observations (GUCC2219)

The GUCC2219 isolate was inoculated on two types of different media, PDA and CMA, and cultured at 25 °C for 15 d. Photographs of the colonies, mycelia, and mycelial structures were taken using a stereomicroscope (Keyence VHX-7000 digital microscope). Sections of the fungal colonies were made with the assistance of a stereomicroscope (Leica S9i) and mounted in water. Photomicrographs of conidiophores and conidia were taken using a compound light microscope (Zeiss Scope 5) equipped with an AxioCam 208 color camera.

### DNA extraction, PCR, and sequencing (GUCC2219)

The GUCC2219 fungal isolate was grown on PDA at 25 °C for 15 d. The resulting mycelia were then scraped off the surface of the plate with a sterile scalpel. Total genomic fungal DNA was extracted using a BIOMIGA Fungus Genomic DNA Extraction Kit (GD2416, BIOMIGA, San Diego, California, USA) following the manufacturer’s protocol. ITS and LSU were PCR-amplified using the primer pair ITS5/ITS4 [53] and LR0R/LR5 [54] (Table 3), respectively. PCRs were conducted in a 25 μl reaction mixture containing 10 μl 2 × Bench Top Taq Master Mix (Biomiga, AT1201, China), 7 μl ddH_2_O, 1 μl forward and reverse primers (10 μM/μl), and 1 μl DNA template. PCR products were commercially sequenced by using the same PCR primers used in the amplification reactions by SinoGenoMax (Beijing).

**Table 3:** Primers and PCR protocols

Target DNA	Primer	Primer sequence 5′–3’	PCR protocol
LSU rDNA	LR0R LR5	ACCCGCTGAACTTAAGC TCCTGAGGGAAACTTCG	95 °C for 3 min; 31 cycles of 94 °C for 30 s, 50 °C for 45 s, and 72 °C for 1 min 30 s; 72 °C for 7 min; and 4 °C on hold
ITS rDNA	ITS5 ITS4	GGAAGTAAAAGTCGTAACAAGG TCCTCCGCTTATTGATATGC	95 °C for 3 min; 31 cycles of 94 °C for 1 min, 50 °C for 45 s, and 72 °C for 3 min; 72 °C for 7 min; and 4 °C on hold

Note: The primers were synthesized by SinGenoMax (Beijing)

### Phylogenetic analysis (GUCC2219)

Sequences of each gene generated from forward and reverse primers were assembled with BioEdit v.7.2.5 [55], and consensus sequences were then combined with related sequences downloaded from National Center for Biotechnology Information (NCBI) (Table 4). Each gene dataset was aligned separately using Mafft v7.187 [56] and manually aligned where necessary. A phylogenetic tree was constructed based on the ITS and LSU sequences as a concatenated dataset using the maximum likelihood (ML) method at the CIPRES web portal [57]. ML was performed using “RAxML-HPC BlackBox” tool [58]. Trees were sampled every 100 generations, and runs were stopped automatically when the average standard deviation of split frequencies fell below 0.01. A 50% majority rule consensus tree was summarized after discarding the first 25% samples. The resulting tree was visualized in FigTree v1.4.3 [59] and by Adobe Illustrator CC 2019.

**Table 4:** GenBank accession numbers of strains used in the phylogenetic analysis

Species	Strain no.	GenBank no.	
		ITS	LSU
*Volutella ciliata*	CBS 483.61	KM231770	KM231635
*V. ciliata*	KNU-516	KM267564	–
*V. citrinella*	DAOM:226720	HQ897821	HQ843771
*V. delonicis*	MFLU 19-1384	NR_171101	NG_073864
*V. lini*	CABI:IMI92688	JQ647452	–
*V. lini*	CABI:IMI224502	JQ693169	–
*V. ramkumarii*	CABI IMI136704	JQ647453	–
*V. thailandensis*	MFLUCC 16-0366	MH388368	MH376742
*Calostilbe striispora*	CBS 133491	KM231789	KM231653

Note: “-” represent the GenBank number doesn‘t exist

### In vitro predatory activity of the fungal isolates against nematodes

In the preliminary screening, a 5 mm-diameter disc of mycelium was taken from the margin of the fungal isolates, transferred to the center of a Petri dish containing 1% WA, and then incubated in the dark at 25 °C for 2 weeks. After the incubation period, Petri dishes were inoculated with a 1 ml nematode suspension containing 1000 nematodes. The nematode suspension was divided into 4-5 drops and spread uniformly inside the periphery of the fungal colonies. Plates without fungi served as controls. The morphology and the average size of the hyphal rings were observed and recorded. Four replicates of each isolate were utilized. The number of nematodes captured in the hyphal rings was counted using a stereomicroscope (Leica S9i, Germany). Microscopic images were captured with a compound light microscope (Zeiss Scope 5) equipped with an AxioCam 208 color camera.

### Nematicidal activity of the fungal fermentation filtrate (GUCC2219)

A 100 μl suspension of nematodes containing approximately 100 nematodes was placed into wells of a 96-well culture plate containing different concentrations (100, 20, 10%) of the fermentation broth. Control wells contained distilled water. The plate was incubated at 28 °C for 72 h. Nematodes were microscopically monitored after 12, 24, 48, and 72 h. At each timepoint, the nematodes were washed and transferred into distilled water to observe their motility as an indication of nematicidal activity. Nematodes were considered dead when they remained immotile upon probing with a fine hair needle, and percentage mortality was calculated. Four replicates were analyzed for each concentration and for the control.

### Data analysis

The data were subjected to two-way analysis of variance (ANOVA), with concentration and posttreatment time (exposure period) serving as the main treatment effects and concentration x time as the interaction. Significant differences between means were determined at *P* < 0.05 using Duncan’s multiple range. All statistical analyses were conducted in MS Excel and SPSS statistics (version 19.0) software. Figures were generated using Origin 2018.

## Supplementary Information

**Additional file 1: Schedule 1**. Some genera or species distributed by nematophagous fungi.

## Data Availability

The datasets used and/or analyzed during the current study are available from the corresponding author on reasonable request.

## Abbreviations

PPNs: Plant parasitic nematodes
PCNs: Potato cyst nematodes
ITS: Internal transcribed spacer
LSU: Large subunit
PCRs: polymerase chain reactions
BLAST: Basic Local Alignment Search tool
PDA: Potato dextrose agar
CMA: Cornmeal agar
WA: Water agar
PDB: Potato dextrose broth
WP: Wettable powder
DP: Dustable powder
NCBI: National Center for Biotechnology Information
ANOVA: Analysis of variance
