# Supplementary material for: A new strain of Volutella citrinella with nematode predation and nematicidal activity, isolated from the cysts of potato cyst nematodes in China
Source: BMC Microbiol. 2021 Nov 22;21:323. doi: 10.1186/s12866-021-02385-x (PMC8607719; doi:10.1186/s12866-021-02385-x)
Supplement: Supplementary file 1 — Additional file 1: Schedule 1. Some genera or species distributed by nematophagous fungi. [file 12866_2021_2385_MOESM1_ESM.docx]

**Schedule 1.** Some genera or species distributed by [nematophagous fungi](https://xueshu.baidu.com/usercenter/paper/show?paperid=1n620vr05f490p709t5g04g0tk240576&site=xueshu_se).

| **[Nematophagous fungi](https://xueshu.baidu.com/usercenter/paper/show?paperid=1n620vr05f490p709t5g04g0tk240576&site=xueshu_se)（Genera or Species）** | | | |
| --- | --- | --- | --- |
| **Nematode-trapping fungi** | **Endoparasitic fungi** | **Toxic fungi** | **Opportunistic fungi** |
| *Arthrobotrys*,  *Cystopage*,  *Candelabrella*,  *Dactylaria*,  *Dactylella*,  *Dactylellina*,  *Didymozoophaga*,  *Drechslerella*,  *Duddingtonia*,  *Gamsylella*,  *Genicularia*,  *Hyphoderma*,  *Monacrosporiella*, *Nematophagus*,  *Stylopage*,  *Triposporina*,  *Trichothecium*,  *Woroninula*,  etc. | *Drechmeria*,  *Hirsutella*,  *Verticillium*,  *Harposporium*,  *Nematoctonus*,  *Haptoglssa*,  *Myzocytium*,  *Catenaria anguillulae*,  *Ophiocordyceps*,  *Tolypocladium*,  *Purpureocillium*,  *Polycephalomyces*,  *Acrostalagmus*,  etc. | *Tolypocladium*,  *Trichoderma*,  *Paecilomyces*,  *Pleurotus*,  *Coprinus*,  *Drechmeria*, *Coniospora*, *Harposporium*, *Anguillulae*, *Lecanicillium*, *Purpureocillium*, *Pochonia*,  *Metapochonia*,  *Catenaria*,  etc. | *Fusarium solani*,  *Fusarium oxysporum*, *Paecilomyces lilacinus*, *Verticillium lecanii*, *Cylindrocarpon destructans*, *Cylindrocarpon* *gracile*, *Gliocladium roseum*, *Stagonospora heteroderae*, *Catenaria auxiliaris*,  *Nematophthora gynophila*,  *Pochonia*,  *Chlamydosporia*,  *Purpureocillium*,  *Drechmeria*,  *Hirsutella*,  *Metapochonia*,  *Verticillium chlamydosporium*  etc. |
